# Psychometric properties of the Arabic version of the Oviedo Grit Scale (A-EGO) in non-clinical adults from the general population

**DOI:** 10.1186/s12888-022-04466-1

**Published:** 2022-12-15

**Authors:** Feten Fekih-Romdhane, Álvaro Postigo, Covadonga González-Nuevo, Mariam Dabbous, Diana Malaeb, Sahar Obeid, Souheil Hallit

**Affiliations:** 1 The Tunisian Center of Early Intervention in Psychosis, Department of Psychiatry “Ibn Omrane”, Razi Hospital, 2010 Manouba, Tunisia; 2 Faculty of Medicine of Tunis, Tunis El Manar University, Tunis, Tunisia; 3 Department of Psychology, University of Oviedo, Oviedo, Spain; 4 School of Pharmacy, Lebanese International University, Beirut, Lebanon; 5 College of Pharmacy, Gulf Medical University, Ajman, United Arab Emirates; 6 School of Arts and Sciences, Social and Education Sciences Department, Lebanese American University, Jbeil, Lebanon; 7 School of Medicine and Medical Sciences, Holy Spirit University of Kaslik, P.O. Box 446, JouniehJounieh, Lebanon; 8 Applied Science Research Center, Applied Science Private University, Amman, Jordan; 9 Research Department, Psychiatric Hospital of the Cross, Jal Eddib, Lebanon

**Keywords:** Grit, Oviedo Grit Scale, Arabic, Validation, Psychometric properties

## Abstract

**Background:**

Given the high clinical utility of grit, and the lack of measures that assess this construct among the Arabic-speaking populations, we propose to examine the psychometric properties of an Arabic translation of the Oviedo Grit Scale (A-EGO) in terms of internal consistency, factor structure, convergent validity, and measurement invariance in a sample of Lebanese adults from the general population.

**Methods:**

We performed a cross-sectional, online study among 575 participants from the Lebanese general population (mean age = 26.28 years [*SD*: 8.83], 72.7% females). The unidimensional factorial structure of the A-EGO was analyzed by confirmatory factor analysis (CFA) using the MPlus8 program. The mean and variance adjusted weighted least squares (WLSMV) was used as the estimation method. The reliability of the scores, the evidence of validity in terms of measurement invariance and correlation with other variables were analyzed.

**Results:**

The present results show that the 10 items of the A-EGO loaded on one factor. The internal consistency was excellent in our sample, as evidenced by a Cronbach’s alpha value of .93. Multi-group confirmatory factor analyses demonstrated invariance across gender, governorate, and marital status at the configural, metric and scalar levels. Additionally, A-EGO scores showed moderate and positive correlations with self-control (*r* = .442), conscientiousness (*r* = .478), and productiveness facet (*r* = .506), supporting the convergent validity of the A-EGO.

**Conclusion:**

Our findings indicate that the A-EGO has good psychometric properties and can be recommended for the assessment of grit among the broader Arabic-speaking people worldwide. Making this scale available in the Arabic language will hopefully foster research in this area in Arab countries.

## Background

Historically, researchers have been concerned with understanding people's reasons for succeeding more than others who show the same talents. One plausible explanation is based on non-cognitive domains [1], including grit [2]. Grit has been defined by Duckworth et al. as: “working strenuously toward challenges, maintaining effort and interest over years despite failure, adversity, and plateaus in progress” [2]. Individuals with high grit exhibit certain traits, such as passion and perseverance, which allow them to achieve their goals beyond aptitudes, opportunities, and even when controlling for conscientiousness trait [2]. According to Duckworth et al., the difference between grit and conscientiousness consists of stamina, or the ability to sustain interest and effort in long-term projects. As such, gritty people do not deviate from their long-term goals, even when there is no positive feedback [3]. For all these reasons, grit has consistently been considered as a positive trait that is positively related to positive psychological and life outcomes (such as academic performance [4], academic achievement [5], hope, mindfulness [6], positive affect, happiness, and life satisfaction [7]), and inversely associated with negative psychological indicators (such as depression [8], anxiety [9], negative affect [7], and suicidal thoughts and behavior [10–12]).

While the grit concept and its association with major aspects of life (including academic performance) have attracted growing research interest in recent years, we could find no previous studies evaluating grit in Arabic-speaking populations living in Arab or non-Arab countries. A cross-cultural meta-analysis published in 2022, encompassing 137 studies on the association between grit and academic achievement, revealed that this relationship remained stable across cultures (individualism versus collectivism) [13]. This suggests that grit would have the same positive impact on people’s lives and outcomes in the Arab cultural backgrounds. Therefore, exploring the grit construct and the ways it relates to positive and negative psychology indicators is a highly relevant research endeavor. This may be particularly meaningful and useful in Arab countries, where youth are facing huge challenges during the last years as shown through raising rates of psychological distress [14], collective violence [15], lack of opportunities, illegal migration [16], unemployment, and school dropout (only 25% of eligible Arab youth are enrolled in a tertiary education) [17].

Despite extended research, there is still no consensus about the definition or measurement of grit [18]. It has initially been conceptualized as a multidimensional construct involving two dimensions, i.e. consistency of interests, and perseverance of effort; and the self-report 12-item two-factor Grit Scale has been developed and validated by Duckworth et al. to illustrate these dimensions [2]. The scale has been then shortened to 8 items while keeping the two-factor solution [3]; and has been validated in different countries and contexts (e.g., Korean [19], Japanese [20], Chinese [21], Russian [22], German [23], and Spanish [24]). However, the conceptualization of the Grit Scale has been criticized later by some authors due to the high overlap with other variables that are supposed to reflect different constructs (i.e., conscientiousness, self-efficacy, self-control, and motivation) [18, 25, 26]. The Grit Scale has also raised debates about its dimensionality, with some findings rather supporting a single-factor structure (e.g., [27, 28]). Additionally, the reliability of the Grit Scale, one of the crucial psychometric properties, has been recently questioned [29].

To overcome these identified gaps, Postigo et al. developed and validated a new grit instrument, the Oviedo Grit Scale (or EGO, a Spanish acronym for “Escala Grit de Oviedo”), in a sample of Spanish-speaking population [18]. Given the high clinical utility of grit, and the lack of measures that assess this construct among the Arabic-speaking populations, the need to validate this new and psychometrically sound scale in the Arabic language becomes obvious. For this, we propose to examine the psychometric properties of an Arabic translation of the Oviedo Grit Scale in terms of internal consistency, factor structure, discriminant validity, and measurement invariance in a sample of Lebanese adults from the general population. We hypothesize that the Arabic Oviedo Grit Scale (A-EGO) would yield a unidimensional factor structure, adequate reliability and evidence of validity, as well as invariance across different groups (based on sex, marital status, and governorate).

## Methods

### Procedure

This was a cross-sectional study, conducted between May and July 2022, enrolling participants residing in Lebanon, from all Lebanese governorates (Beirut, Mount Lebanon, North, South, and Bekaa). Our sample was chosen using the snowball technique; a soft copy of the questionnaire was created using google forms software, and an online approach was conceived to proceed with the data collection. The study’s main aims and goals, in addition to instructions for filling the questionnaire, were conveyed online for the participants, prior to participation. The participants did not receive any reward for participating in the study. Anonymity and confidentiality were carefully respected, and the strict fulfillment of data protection was guaranteed. Later, initial participants were asked to recruit other participants they know, preferably as diverse as possible regarding place of habitat within the Lebanese governorates, gender and age. The average response time of the participants to fill the questionnaire was 10–15 min.

### Participants

The sample comprised 575 participants enrolled conveniently from the Lebanese general population. The age ranged between 18 and 66 years, with a mean of 26.28 ± 8.83 years, with 72.7% of the sample women, 78.3% single, and 50.3% from rural areas.

### Instruments

#### ***Oviedo grit scale (EGO; ***[18***])***

The EGO is a unidimensional questionnaire of 10 items that evaluates grit (e.g., “Although the results seem far off, I persist in the task”). The items are scored on a Likert scale from 1 (totally disagree) to 5 (totally agree). All items were formulated in a positive direction to reduce response bias [30]. The instrument has excellent reliability (α = 0.94) as well as good evidence of convergent validity [18]. The adaptation of the A-EGO followed the guidelines of the International Test Commission [31–33]. The Spanish version of the EGO, as well as all other scales, were forward and back translated by two certified translators. Forward translation was first conducted by a single bilingual translator, whose native language is Arabic and fluent in English. An expert committee formed by healthcare professionals and a language professional verified the Arabic-translated version. A backward translation was then performed by a native English speaker translator, fluent in Arabic and unfamiliar with the concepts of the scales. The back-translated English questionnaire was subsequently compared with the original English one, by the expert committee, aiming to discern discrepancies and to solve any inconsistencies between the two versions. No discrepancies were noted in terms of intellectual consistency [34].

#### ***Brief self-control scale*** (BSCS; [35])

The BSCS is a 13-item questionnaire that evaluates self-control (e.g., “I am good at resisting temptation”). Items are scored on a Likert scale from 1 (not at all like me) to 5 (very much like me). As per the recommendation of Lindner et al. [36], the total score of the questionnaire was used in the present study, with an adequate reliability (α = 0.81).

#### ***Big five inventory 2 (BFI-2; ***[37]***)***

The BFI-2 is composed of 60 items, which measure the Big Five personality domains and 15 more specific facets. The items are scored on a Likert scale from 1 (strongly disagree) to 5 (strongly agree). In this line, the BFI-2 evaluates three facets (four items per facet) for each of the big five personality domains. The Arabic translation was directly obtained by Dr Soto. For the present study, the five domains of the Big Five were used, in addition to the facets of conscientiousness, as it is the trait most closely related to grit. Reliability values (α) for each of the variables were as follows: Conscientiousness (domain): α = 0.86; Organization: α = 0.75; Productiveness: α = 0.63; Responsibility: α = 0.64; Negative emotionality (domain): α = 0.74; Extraversion (domain): α = 0.68; Agreeableness (domain): α = 0.78; Open-Mindedness (domain): α = 0.73.

### Data analysis

First, descriptive statistics were done (mean, standard deviation, skewness and kurtosis) for the 10 items of the A-EGO. The discrimination indices of the items were analyzed (corrected item-test correlations); they are considered adequate at values over 0.20 [38].

Second, the unidimensional factorial structure of the A-EGO was analyzed by confirmatory factor analysis (CFA). Some items showed values of skewness and kurtosis outside the range of ± 1, which is why the CFA was performed on the polychoric correlation matrix [39]. The mean and variance adjusted weighted least squares (WLSMV) was used as the estimation method. The following indices of fit were computed: comparative fit index (CFI), Tucker-Lewis index (TLI), and root mean square error of approximation (RMSEA). A good fit was considered established when CFI and TLI were above 0.95 and RMSEA below 0.08 [40]. Evidence of convergent validity was studied by means of Composite Reliability (CR), and Average Variance Extracted (AVE) when higher than 0.50.

In addition, given the importance of studying the factor structure of a construct through different populations, the measurement invariance was studied in terms of sex, governorate, and marital status. For this, the configural, metric, and scalar invariance were studied through multi-group confirmatory factor analysis (MG-CFA). When dealing with nested models, measurement invariance was considered reached if ΔCFI < -0.01 and ΔRMSEA < 0.015 [41].

Finally, to evaluate evidence of validity in relation to other variables (AERA, APA, NCME, 2014), Pearson’s test was used to correlate the A-EGO score with the self-control and Big Five model (domains and facets of conscientiousness) scores.

Descriptive statistics, Pearson’s correlations, reliability of the scores, and differences between groups were calculated with the SPSS program version 24 [42]. The CFA and measurement invariance were carried out with the MPlus8 program [43].

## Results

First, descriptive statistics of the items of the A-EGO were studied. Each of the items showed adequate values in skewness and kurtosis (Table 1). Item one showed a slightly high kurtosis value. The discriminative power was very high for each item (I.D. [0.636—0.776]).

**Table 1:** Descriptive Statistics, Discrimination Indices, and Factor Loadings of the Items on the A-EGO

Item	Mean	Standard Deviation	Skewness	Kurtosis	Item-test correlation (corrected)	Factor loading
01	4.14	0.838	-1.391	3.043	.756	.848
02	4.04	0.800	-1.012	1.847	.731	.826
03	3.96	0.860	-0.722	0.385	.636	.726
04	4.04	0.873	-0.918	0.847	.745	.836
05	4.03	0.817	-0.903	1.186	.694	.776
06	4.00	0.828	-0.775	0.788	.734	.821
07	4.11	0.850	-0.990	1.077	.758	.854
08	4.00	0.861	-0.870	0.916	.746	.827
09	4.13	0.817	-1.062	1.657	.776	.867
10	3.96	0.947	-1.059	1.130	.691	.776
Total	40.41	6.655	-0.832	1.455	-	-

Second, the CFA results confirmed the unidimensional model of the A-EGO in the total sample (Table 2). Also, the evidence of convergent validity was adequate according to the composite reliability (CR = 0.95), and average variance explained (AVE = 0.73). Next, the invariance measurement was studied based on sex, governorate, and marital status, with the three studied levels of invariance (configural, metric and scalar) being fulfilled, as shown in Table 2. In addition, the reliability of the A-EGO was very high (α = 0.93).

**Table 2:** Factor Structure and Measurement Invariance for the A-EGO Based on Sex, Governorate, and Marital Status

	CFI	TLI	RMSEA [90%]	ΔCFI	ΔRMSEA
Total	.986	.982	.086 [.074 - .098]	-	-
Sex					
Men	.988	.985	.094 [.069 - .120]	-	-
Women	.986	.983	.089 [.075 - .104]	-	-
Configural	.987	-	.090 [.077 - .103]	-	-
Metric	.988	-	.080 [.068 - .093]	.001	-.010
Scalar	.992	-	.056 [.044 - .067]	.004	-.024
Governorate					
Urban	.987	.984	.089 [.071 - .108]	-	-
Rural	.985	.981	.097 [.080 - .115]	-	-
Configural	.986	-	.093 [.081 - .106]	-	-
Metric	.988	-	.084 [.072 - .096]	.002	-.009
Scalar	.991	-	.062 [.050 - .073]	.003	-.022
Marital status					
Single	.992	.990	.071 [.057 - .086]	-	-
Married	.973	.965	.136 [.109 - .164]	-	-
Configural	.989	-	.089 [.076 - .102]	-	-
Metric	.991	-	.073 [.061 - .086]	.002	-.016
Scalar	.993	-	.056 [.045 - .068]	.002	-.017

Finally, in terms of evidence of validity in relation to other variables, Table 3 provides Pearson’s correlations between grit, measured through the A-EGO, and the remaining variables. A-EGO scores showed moderate relations with self-control, and the domain of conscientiousness and its facets, especially productiveness. In the same way, the A-EGO was negatively correlated to negative emotionality.

**Table 3:** Pearson’s Correlations between the A-EGO and the BSCS, and the BFI-2

	A-EGO
BSCS	
Self-Control	.442
BFI-2	
Conscientiousness	.478
Organization (facet)	.335
Productiveness (facet)	.506
Responsibility (facet)	.417
Negative Emotionality	-.267
Extraversion	.398
Agreeableness	.314
Open-Mindedness	.337

## Discussion

Grit is a clinically relevant psychological construct that has been recognized as positive and protective due to its consistent association with a wide range of positive outcomes. Although grit has received increased research attention across different parts of the world, no studies have emerged from Arab countries so far. This is mainly due to a lack of valid measures of the construct in the Arabic language. For these reasons, we aimed through the present study to investigate the psychometric properties of the Arabic version of a theoretically-based and psychometrically solid grit scale, i.e., the EGO [18]. As expected, the present results show that the 10 items loaded into one factor. The A-EGO also demonstrated excellent internal consistency, good convergent validity, and measurement invariance across gender, marital status, and governorate. These findings suggest that the A-EGO is a valid and reliable self-report grit measure among Arabic-speaking participants.

The internal consistency of the A-EGO was excellent in our Arabic-speaking Lebanese adults sample, as evidenced by a Cronbach alpha value of 0.93. In the original validation study, the EGO has proven psychometrically advantageous over other grit scales by demonstrating an excellent reliability in Spanish, healthy community-based adults [18]. Evidence for adequate reliability has also been shown in other Spanish [44] and Portuguese [45] samples. Furthermore, our findings confirmed the one-factor structure proposed by the developers of the instrument [18], supporting that the grit construct should better be considered as unidimensional. In addition, the unidimensional structure of the original EGO remained unchanged across age and gender groups [18]. This structural stability across different populations further confirms its unidimensionality [46]. Consistently, multi-group confirmatory factor analyses demonstrated invariance across gender, governorate, and marital status at the configural, metric and scalar levels in our Lebanese sample. Therefore, the A-EGO can be used to make psychometrically sound comparisons between individuals with different characteristics (males versus females, urban versus rural, married versus unmarried).

As hypothesized, we found that the A-EGO showed moderate and positive correlations with measures of self-control, conscientiousness (especially with productiveness facet), as well as moderate and negative correlations with negative emotionality. This suggests, in agreement with the literature, that grit, self-control, and conscientiousness are distinct constructs, albeit with some similar features [18]. Conscientiousness implies a timely tasks completion, whereas grit refers to a future-oriented capacity to maintain consistency in accomplishing one’s goals and objectives [2, 18]. Self-control is distinct from grit by the fact that it refers to the ability to delay gratification [47], which reflects a short-term behavior rather than a long-term consistent interest [18]. Further studies are still needed to clarify and deepen our understanding of the differences between these concepts.

Given all that, the present study shows that the EGO scale is a reliable tool with adequate evidence of validity for use in Arabic-speaking countries. Thus, an instrument that allows the assessment of perseverance and passion for long-term goals has been validated in Lebanon. The EGO scale can be applied in various contexts where grit has shown a strong relationship, such as the educational context [47, 48], health [49] and work context [43], among others. Future studies should aim at studying whether the EGO scale works correctly as a general domain in Arabic-speaking countries or, on the contrary, whether it is convenient to adapt the scale to the assessment of grit as a specific domain (e.g., educational context, study academic grit instead of general grit).

In sum, we contribute to existing literature by providing the first valid grit measure in the Arabic language. Given that academic achievement is one of the most important and consistently reported correlate of grit among student populations across cultures [13], we hope that providing the A-EGO will be helpful to school counselors and educational practitioners in Arab settings who are dealing with higher rates of school dropout, as well as lower rates of commitment to school and learning than the world average [48]. We also hope that offering a valid and reliable grit scale to Arab researchers will contribute to emerging research on grit in the Arab region.

### Study limitations

The present findings are valuable in terms of better understanding of the cross-national utility of the EGO and allowing future research in this area in the non-previously studied Arabic-speaking populations. However, some limitations need to be discussed. The study has a cross-sectional design, which does not allow for causal inferences. In addition, due to its web-based and self-report nature, the study may be subject to social desirability bias and selection bias. Finally, test–retest reliability has not been tested in the present validation study, and need to be subject of future research to examine the stability of A-EGO scores across time.

## Conclusions

Our findings indicate that the A-EGO has good psychometric qualities, and can be recommended for use to assess grit among Arab individuals. In addition, given its previously demonstrated cultural stability, we suggest that the A-EGO is appropriate for use among the broader Arabic-speaking people all over the world. Additional cross-cultural research involving the unexplored Arab countries are needed to confirm these suggestions. Making this scale available in the Arabic language will hopefully foster research in this area in Arab countries, and benefit the struggling youth in these contexts. Given the power that the study of the grit trait has had in the last decade, having a reliable and valid instrument for its evaluation in Arabic-speaking countries will be a good starting point to study its influence in different contexts of people's lives.

## Data Availability

All data generated or analyzed during this study are not publicly available due to restrictions from the ethics committee. The dataset supporting the conclusions is available upon request to the corresponding author.
